# Digital pain extent is associated with pain intensity but not with pain-related cognitions and disability in people with chronic musculoskeletal pain: a cross-sectional study

**DOI:** 10.1186/s12891-022-05700-3

**Published:** 2022-07-30

**Authors:** Alejandro Luque-Suarez, Deborah Falla, Marco Barbero, Consolacion Pineda-Galan, Derboni Marco, Vincenzo Giuffrida, Javier Martinez-Calderon

**Affiliations:** 1 Facultad de Ciencias de La Salud, Departamento de Fisioterapia, Universidad de Malaga, Malaga, Spain; 2 Instituto de Investigación Biomédica de Málaga (IBIMA), Malaga, Spain; 3 Centre of Precision Rehabilitation for Spinal Pain (CPR Spine), School of Sport, Exercise and Rehabilitation Sciences, College of Life and Environmental Sciences, University of Birmingham, Birmingham, UK; 4 Rehabilitation Research Laboratory 2rLab, Department of Business Economics, Health and Social Care, Sciences and Arts of Southern Switzerland, University of Applied, Manno/Landquart, Switzerland; 5 Istituto Dalle Molle Di Studi Sull’Intelligenza Artificiale (IDSIA), Scuola Universitaria Professionale Della Svizzera Italiana (SUPSI), Università Della Svizzera Italiana (USI), Lugano, Switzerland

**Keywords:** Chronic pain, Musculoskeletal pain, Cognition, Digital pain extent, Cross-sectional, Pain drawing

## Abstract

**Background:**

To evaluate whether digital pain extent is associated with an array of psychological factors such as optimism, pessimism, expectations of recovery, pain acceptance, and pain self-efficacy beliefs as well as to analyse the association between digital pain extent and pain intensity and pain-related disability in people with chronic musculoskeletal pain.

**Methods:**

A descriptive cross-sectional study conducted in a primary health care setting was carried out including 186 individuals with chronic musculoskeletal pain. Patient-reported outcomes were used to assess psychological factors, pain intensity, and pain-related disability. Digital pain extent was obtained from pain drawings shaded using a tablet and analysed using novel customized software. Multiple linear regression models were conducted to evaluate the association between digital pain extent and the aforementioned variables.

**Results:**

Digital pain extent was statistically significantly associated with pain intensity. However, digital pain extent was not associated with any psychological measure nor with pain-related disability.

**Discussion:**

The results did not support an association between digital pain extent and psychological measures.

## Background

Globally, the financial and societal burden resulting from chronic musculoskeletal pain is substantial. Many chronic musculoskeletal pain disorders are degenerative and irreversible and may increase in prevalence as a consequence of aging [1, 2]. Currently, chronic musculoskeletal pain is the second leading cause of years lived with disability [3], and the associated direct and indirect costs are considerable across countries [4]. However, the low mortality rate [5] often leads to musculoskeletal disorders being underappreciated and underestimated by health care providers and policymakers.

Widespread pain, commonly defined as a pain characterized by multiple painful sites, is a common symptom among individuals with chronic pain [6, 7]. It is considered as a common proxy to central sensitization [8], which is a neurophysiological phenomenon that encompasses neurobiological changes in dorsal horn neurons such as increased excitability, strengthened synaptic transmission, and reduced inhibition [9]. Central sensitization can be provoked by both peripheral and central drivers (psychological, behavioural and social factors) [9]. Although psychological factors and central sensitization are distinct, they can have a direct influence on the nervous system [9], and, thus, their role must be considered.

Widespread pain can be estimated using a validated pain site checklist [10, 11], or more recently by the digitalization of pain drawings. Notably, digital pain drawing allows higher accuracy in defining the pain distribution and moreover the quantification of “pain extent” [12], a measure that cannot be performed using the traditional paper and pencil approach. The digital pain extent, as an intrinsic characteristic of somatic pain is usually expressed as a percentage of the area of a body chart [10], has been positively associated with pain intensity in different populations including low back pain [13], neck pain [13], fibromyalgia [14], migraine [15] as well as musicians with playing-related musculoskeletal disorders [16]. Some observational studies have also shown that a larger digital pain extent is associated with physical disability and negative psychological states such as depression in people with chronic neck pain [17] or chronic whiplash-associated disorders [18]. Psychological factors are key in the onset and maintenance of persistent pain (e.g., musculoskeletal pain) [19]. However, systematic reviews have found that the association between different methods of pain extent evaluation and psychological factors (e.g., depression) is weak (strength of the evidence) or inconclusive ( both positive and negative associations) [20, 21].

Pain research have mainly focused on evaluating the association between pain extent and negative psychological states such as anxiety, distress, hypochondriasis, hysteria, kinesiophobia, pain catastrophizing, and somatization [20, 21]. Nevertheless, the role that psychological protective factors (i.e., general self-efficacy) play in how people with chronic pain perceive the extension of that pain undergone limited evaluation [20, 21]. Resilience, a psychological protective factor, has been associated with widespread pain sensitivity in participants with shoulder pain [8].Expectations of recovery, optimism, pain acceptance, and pain self-efficacy are involved in widespread pain conditions such as fibromyalgia [22–25]. To our knowledge, no observational studies have analysed the potential association between these psychological protective factors and digital pain extent in chronic musculoskeletal pain.

This cross-sectional study sought to evaluate whether digital pain extent is related to optimism, pessimism, expectations of recovery, pain acceptance, and pain self-efficacy in people with chronic musculoskeletal pain. The potential association between digital pain extent and pain intensity and pain-related disability has been also analysed. A larger digital pain extent was hypothesised to be associated with more pessimism and lower levels of optimism, expectations of recovery, pain acceptance, pain self-efficacy beliefs. Additionally, a greater digital pain extent was also hypothesised to be related to higher levels of pain intensity and pain-related disability. This study may provide both clinicians and researchers a new insight on the relationships that some protective psychological factors may have on pain extent in people with chronic MSK pain.

## Methods

### Study design

This cross-sectional study followed the same methodology as a previous cross-sectional study which evaluated the role that psychological protective factors play in chronic pain intensity and pain interference [26]. Ethical approval was granted by the local Ethics Committee. Detailed information about study design, participants and setting, and eligibility criteria can be consulted elsewhere [26].

### Participants

Briefly, individuals with a diagnosis of chronic musculoskeletal pain (MSK) pain [27] were recruited from four primary care centers in the province of Malaga, Spain, from September 2017 to December 2018. Inclusion criteria: (i) people with a diagnosis of chronic MSK pain [27]; aged 18 or older. Exclusion criteria: a history of musculoskeletal trauma (e.g., fracture); [ii] postoperative musculoskeletal pain during the previous six months; [iii] musculoskeletal pain suspected to be originated from neurological (e.g., stroke), neoplastic (e.g., breast cancer) and/or referred pain (e.g., visceral referred pain) and; [iv] participants unable to provide written informed consent.

### Variables

Outcome measures (Dependent variables).

The dependent variables are described as follows:

1. Chronic pain intensity and pain-related disability: they were evaluated by the Chronic Pain Grade Scale (CPGS), Spanish version [28]. This questionnaire presents two subscales (pain intensity and pain interference), with both ranging from 0–30. Higher scores reflect more pain intensity and more pain interference (disability).
2. Expectations of recovery were assessed using the following self-reported tool: the Expectations for Complementary and Alternative Medicine Treatments (EXPECT) [29]. This self-reported tool was designed to evaluate expectations associated with treatment, presenting high internal consistency and moderate test–retest reliability in people with chronic pain [29].
3. Pain acceptance was assessed by the 20-item Chronic Pain Acceptance Questionnaire (CPAQ), Spanish version [30]. Values range from 0 to 120, with lower scores showing less pain acceptance.
4. Optimism and pessimism were evaluated by the 10-item version of the Life Orientation Test-Revised (LOT-R), Spanish version [31], with higher values reflecting more optimism and pessimism.
5. Pain self-efficacy was measured by the 10-item Pain Self-Efficacy Questionnaire (PSEQ*)* [32]. The total score can range from 0 to 60, with higher scores reflecting more pain self-efficacy.

More information about these dependant variables are reported elsewhere [26].

### Independent variables

The independent variables were digital pain extent (independent variable of interest), gender, age, height, duration of symptoms, comorbidity, educational level, current treatment, and professional status (covariate). Most of the aforementioned covariates were chosen based on their potential role shown in previous studies [33]. Specifically, digital pain extent was evaluated as follows: participants were instructed to report their pain extent using a tablet (iPad Air2, Apple computer, CA, USA). A custom web application (Pain Sketch V2.0.6) was installed on the tablet to provide the possibility of sketching the pain areas on four different body charts (frontal male view, dorsal male view, frontal female view, dorsal female view). The pain drawings were completed using a stylus pen with a standardized stroke. Participants were asked to shade, as precisely as possible, their usual pain regardless of the type and intensity of pain. Digital pain extent was computed for each digital body chart as the sum of the pixels and expressed as a percentage of the total body chart area. Pain frequency maps were also created to illustrate where the participants most frequently reported pain. Any shading outside of the body chart borders was excluded from the analysis. The reliability of the described procedures has been described in previous publications [13, 34].

### Sample size calculation

We estimated a priori a sample size of 180 people with chronic musculoskeletal pain. The calculation was estimated regarding the assumption of 15 individuals per each factor that was considered to be needed to include in the multiple linear regression model (chronic pain intensity, pain-related disability, expectations of recovery, pain acceptance, pain self-efficacy, age, gender, height, comorbidity, educational level, duration of symptoms, current treatment, professional status) [35].

### Statistical analysis

Descriptive analyses and topographic pain descriptions using pain frequency maps were developed. Spearman correlation co-efficients were used to observe potential correlations between digital pain extent and the factors above mentioned (i.e., pain self-efficacy). Factors that significantly correlated with digital pain extent were included in a multiple linear regression model. Gender, age, height, duration of symptoms, comorbidity, educational level, current treatment, and professional status were included as covariates. The ordinary least squares method was applied [36]. A Python script, supported by the *stats model* package was used to perform the data analysis [37]. Statistical significance was set at *P* < 0.05 for all analyses.

## Results

### Baseline demographic and clinical characteristics

A total of 186 individuals with chronic musculoskeletal pain participated (71.5% female). The mean age of the sample was 52.7 (SD 10.6) years. Almost 90% of the sample reported musculoskeletal pain for more than 12 months. Many participants self-reported that low back pain was the main pain location with which they visited a health care professional. Table 1 presents descriptive information about the whole sample.

**Table 1:** Sample characteristics

Variable	Mean (SD)	n (%)
Age (years)	53 (10.6)	
Male		53 (28.5)
Female		133 (71.5)
Height (cm)	165 (10)	
**Education level**		
Very low		8 (4.3)
Low		50 (26.9)
Medium		72 (38.7)
High		47 (25.3)
Very high		7 (3.8)
**Employment status**		
Working		73 (39.2)
Unemployment		28 (15.1)
Sick leave		14 (7.5)
Retirement		30 (16.1)
Housework		39 (21)
**Health status**		
Presence of co-morbidities (yes/no question)		103 (55.4)
**Current treatment**		
No treatment		41 (22)
Pharmacological		18 (9.7)
Injection		0 (0)
Physiotherapy		117 (62.9)
Other treatments		8 (4.3)
**Pain**		
Pain extent of the total body chart area (%)	4.8 (6.5)	
Pain duration, 3–6 months		12 (6.5)
Pain duration, 6–12 months		12 (6.5)
Pain duration, > 12 months		162 (87)
Low back pain		78 (41.9)
Neck pain		43 (23.1)
Shoulder pain		44 (23.7)
Knee osteoarthritis		3 (1.6)
Hip osteoarthritis		8 (4.3)
Fibromyalgia		2 (1.1)
Thoracic pain		2 (1.1)
Wrist pain		1 (0.5)
Ankle pain		5 (2.7)
Elbow pain		1 (0.5)
**PROMs (patient reported outcome measures)**		
GCPS, pain intensity (0–100)	58.3(22.1)	
GCPS, pain-related disability (0–100)	45.2 (30.3)	
PSEQ, pain self-efficacy (0–60)	38.8(15.5)	
EXPECT, expectations of recovery (0–40)	25.9 (10.3)	
LOT-R, optimism (0–12)	7.5 (3.8)	
LOT-R, pessimism (0–12)	4.1 (3.1)	
CPAQ, pain acceptance (0–120)	69.8 (14.1)	

*GCPS* Chronic Pain Grade Scale, *PSEQ* Pain Self-efficacy questionnaire, *LOT-R* Life Orientation Test-Revised, *CPAQ* Chronic Pain Acceptance Questionnaire

**Fig. 1:**
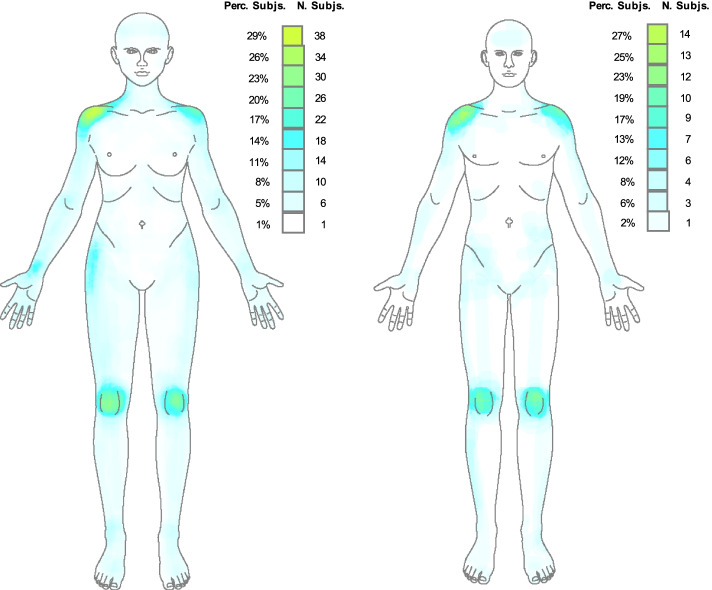
Pain frequency maps by gender (frontal). Perc Subjs: percentage of subjects; Subjs N: number of subjects

**Fig. 2:**
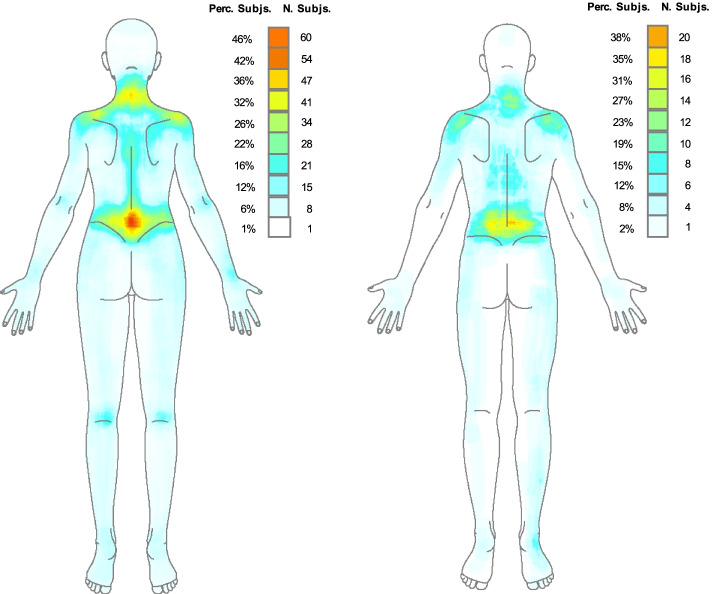
Pain frequency maps by gender (dorsal). Perc Subjs: percentage of subjects; Subjs N: number of subjects

Additionally, the pain frequency maps (frontal and dorsal) by gender (female and male), are presented in Figs. 1 and 2.

### Univariate associations between digital pain extent and psychological factors, pain intensity, and pain-related disability

The correlation analysis showed that a larger digital pain extent was significantly associated with higher pain intensity, pain-related disability, and pain acceptance. Furthermore, a larger digital pain extent was significantly associated with lower pain self-efficacy beliefs (Table 2).

**Table 2:** Univariate associations between digital pain extent and psychological factors, pain intensity, and pain-related disability

Variable	The correlation co-efficient (p)
Digital pain extent	
GCPS, pain intensity (0–100)	0.38***
GCPS, pain-related disability (0–100)	0.33***
PSEQ, (0–60)	-0.18*
EXPECT (0–40)	-0.13
LOT-R, optimism (0–12)	-0.01
LOT-R, pessimism (0–12)	0.03
CPAQ (0–120)	0.09*

*GCPS* Chronic Pain Grade Scale, *PSEQ* Pain Self-efficacy questionnaire, *LOT-R* Life Orientation Test-Revised, *CPAQ* Chronic Pain Acceptance Questionnaire. *: p < 0.05; **: p < 0.01; ***: p < *0.001*

### Multiple linear regression model

The multiple linear regression model evaluating the association between digital pain extent (independent variable of interest), psychological factors and pain-related variables GCPS Pain, GCPS Disability, CPAQ, PSEQ (dependent variables) is presented in Table 3. Gender, age, height, duration of symptoms, comorbidity, educational level, current treatment and professional status were included as covariate. A multiple linear regression model is provided for each dependent variable. Variance inflation factor (VIF) scores were all below “3” showing that there is no significant multicollinearity between any pair of independent variables (Table 4) [38]. A larger digital pain extent was only significantly associated with higher pain intensity after controlling for multiple covariates. The predictive value for each variable of interest was as follows: GCPS-Pain intensity R^2^_adj_ = 0.38; GCPS-Pain-related disability R^2^_adj_ = 0.39; CPAQ R^2^_adj_ = 0.02; PSEQ R^2^_adj_ = 0.14.

**Table 3:** Multiple linear regression model (first line: coefficient; second line: standard error; third line: 95% confidence interval)

	**GCPS Pain**	**GCPS Disability**	**CPAQ**	**PSEQ**
Constant (intercept)	46.90	-60.10	68.48*	-28.27
	(43.85)	(60.50)	(32.44)	(36.29)
	[-39.83, 133.62]	[-180.64, 58.65]	[4.32, 132.65]	[-100.04, 43.50]
Digital pain extent	1.24*	0.85	0.44	-0.38
	(0.48)	(0.67)	(0.36)	(0.40)
	[0.28, 2.19]	[-0.47, 2.17]	[-0.27, 1.15]	[-1.17, 0.41]
Gender	2.95	9.47	0.18	6.17
	(4.66)	(6.42)	(3.45)	(3.85)
	[-6.26, 12.16]	[-3.24, 22.18]	[-6.64, 6.99]	[-1.45, 13.79]
Age	-0.22	-0.27	0.08	0.13
	(0.17)	(0.24)	(0.13)	(0.14)
	[-0.56, 0.12]	[-0.74, 0.20]	[-0.17, 0.33]	[-0.15, 0.41]
Height	9.89	69.70*	-3.67	33.23
	(24.24)	(33.45)	(17.94)	(20.06)
	[-38.06, 57.84]	[3.55, 135.85]	[-39.14, 31.80]	[-6.45, 72.90]
Duration of symptoms	0.98	-7.75	2.06	1.99
	(2.93)	(4.04)	(2.17)	(2.43)
	[-4.82, 6.78]	[-15.75, 0.25]	[-2.23, 6.35]	[-2.81, 6.79]
Comorbidity	-0.19	-0.88	3.25	-0.20
	(2.93)	(4.04)	(2.17)	(2.42)
	[-5.99, 5.60]	[-8.88, 7.11]	[-1.04, 7.54]	[-4.99, 4.60]
Educational level	-8.10***	-9.65***	-2.09	3.02*
	(1.72)	(2.37)	(1.27)	(1.42)
	[-11.50, -4.70]	[-14.34, -4.96]	[-4.61, 0.42]	[0.20, 5.83]
Current treatment				
Pharmacological	7.87	24.01*	-2.91	-8.48
	(5.92)	(8.16)	(4.38)	(4.90)
	[-3.83, 19.57]	[7.87, 40.15]	[-11.57, 5.74]	[-18.16, 1.20]
Physiotherapy	16.11***	30.70***	0.20	-10.38*
	(3.89)	(5.37)	(2.88)	(3.22)
	[8.42, 23.81]	[20.09, 41.32]	[-5.49, 5.90]	[-16.75, -4.01]
Other treatments	13.23	30.08**	-2.67	-4.50
	(7.00)	(9.66)	(5.18)	(5.79)
	[-0.61, 27.07]	[10.98, 49.17]	[-12.92, 7.57]	[-15.96, 6.95]
Employment Status				
Housework	3.97	6.35	1.62	-1.90
	(4.27)	(5.90)	(3.16)	(3.54)
	[-4.48, 12.42]	[-5.31, 18.00]	[-4.62, 7.87]	[-8.89, 5.09]
Retirement	-0.19	14.93*	4.30	-1.92
	(4.76)	(6.57)	(3.52)	(3.94)
	[-9.60, 9.23]	[1.94, 27.93]	[-2.67, 11.26]	[-9.72, 5.87]
Sick leave	23.35**	31.79**	-2.06	-10.50
	(6.24)	(8.61)	(4.62)	(5.16)
	[11.01, 35.69]	[14.77, 48.82]	[-11.19, 7.07]	[-20.71, -0.29]
Unemployment	4.19	3.66	-2.86	-2.50
	(4.60)	(6.35)	(3.41)	(3.81)
	[-4.91, 13.30]	[-8.90, 16.22]	[-9.60, 3.87]	[-10.03, 5.03]

*GCPS* Chronic Pain Grade Scale, *PSEQ* Pain Self-efficacy questionnaire, *CPAQ* Chronic Pain Acceptance Questionnaire

^*^: p < 0.05; **: p < 0.01; ***: p < *0.001*

**Table 4:** Variance inflation factors (VIF) showing the correlation among independent variables. VIF scores were all below 3 indicating that multicollinearity was not an issue

		**VIF**
Digital Pain extent		1,14
Gender		2,25
Age		1,74
Height		1,97
Duration of symptoms		1,17
Comorbidity		1,08
Educational level		1,35
Current treatment		
Other treatments		1,23
Pharmacological		1,48
Physiotherapy		1,79
Professional status		
Housework		1,63
Retirement		1,67
Sick leave		1,21
Unemployment		1,27

## Discussion

This is the first study analysing the association between digital pain extent and some protective psychological factors in a wide sample of mixed chronic musculoskeletal pain conditions. The objective of this cross-sectional study was to analyse the potential associations between digital pain extent and some protective psychological factors as well as pain intensity and pain-related disability in people with chronic musculoskeletal pain. Contrary to our hypothesis, digital pain extent was not associated with any psychological measures or pain-related disability, after a multilinear regression analysis controlling for multiple covariates. Our results, however, did show that people perceiving a larger digital pain extent report higher pain intensity. Previous literature has reported some discrepancies when the association between digital pain extent and pain intensity is examined. For example, Barbero et al. reported that a larger digital pain extent is associated with greater pain intensity in chronic conditions such as fibromyalgia [14], neck pain [13], low back pain [13], and migraine [15]. On the other hand, Fuensalida-Novo et al. [39], Fernandez-de-las-Peñas et al. [40], and Fernandez-de-las-Peñas et al. b [41] found that digital pain extent was not related to pain intensity in episodic cluster headache, episodic migraine, and carpal tunnel syndrome, respectively. This inconsistency between results could be explained by the differing pain mechanisms for each condition. Chronic musculoskeletal conditions are frequently implicated with “nociplastic” pain [42] whereas conditions such as carpal tunnel syndrome is a typical example of neuropathic pain. Similarly, Uthaikhup et al. [15] reported no correlation between digital pain extent and intensity in patients with episodic migraine. Notably, in the same study, a moderate positive correlation was demonstrated in the case of chronic migraine. The temporal persistence of pain symptoms may be another element implicated in the somatic spreading of pain (i.e., pain extent).

Psychological Factors are Involved in the Onset and/or Persistence of Musculoskeletal Pain [19]. Discrepancies have been also reported when the association between pain extent and pain-related cognitions and emotions has been evaluated. For example, Falla et al. [18] concluded that greater self-efficacy beliefs are associated with lesser digital pain extent among individuals with whiplash-associated disorders. However, our study did not find any significant association between digital pain extent and pain self-efficacy in a cohort of mixed chronic musculoskeletal pain disorders. One possible explanation for this difference could relate to the specific measure of self-efficacy. Falla et al. [18] used the Self-Efficacy Scale [43] whereas our study used the Pain Self-Efficacy Questionnaire [44] which just recently, was recommended as the gold standard to assess pain self-efficacy beliefs in people with musculoskeletal disorders [45]. Discrepancies in terms of pain catastrophizing have also been detected. Willett et al. [46] found an association between larger digital pain extent and more pain catastrophizing in people with hip osteoarthritis although this association was not demonstrated in people with whiplash-associated disorders [18]. Additionally, our study and a previous study [18] did not identify associations between digital pain extent and a broad list of pain-related cognitions and emotions such as pain acceptance, optimism, pessimism, expectations of recovery, kinesiophobia, and/or pain catastrophizing.

The comparison of our results with the results from studies using different methodologies to measure pain extent should also be considered. For example, Gerdle et al. [47] analysed spatial extent of pain in a large number of patients with chronic pain (*n* = 39,916). Pain extent was assessed by evaluating the presence of pain in thirty-six predefined anatomical areas. They found an association between pain intensity and pain extent which is consistent with our results. On the contrary, they found associations between anxiety-depression and pain extent, which contrasts to our findings as we have shown a lack of association between digital pain extent and some protective psychological factors. The heterogeneity between methods of evaluation, sample characteristics, psychological variables analysed, and study design likely explain this discrepancy.

### Clinical implications

The number of pain sites reported by people with chronic musculoskeletal pain has potentially relevant implications for clinical practice. Digital assessment of pain extent could have a diagnostic value for identifying somatosensory patterns/profiles related to altered nociception [12]. The value that widespread pain has as an indicator of the psychological status of a person with MSK pain, remains unclear. While some studies have shown some association between them, others have found weak associations [21]. The results of this cross-sectional study do not support its use when the association between digital pain extent and psychological measures is evaluated. This is in line with previous systematic reviews analysing this association [20, 21]. Based on our results, we cannot conclude that a widespread pain in a patient with chronic MSK pain can alert clinicians to consider a psychological screening.

### Future research

A previous scoping review showed that digital pain extent is more associated with patient-reported outcomes among individuals with persistent musculoskeletal pain rather than other chronic pain conditions such as migraine. However, our sample included people with chronic musculoskeletal pain only and a lack of association was found for most of the variables of interest. To further examine this discrepancy, future studies should: (i) evaluate the association between digital pain extent and a broad list of biopsychosocial factors in people with persistent musculoskeletal pain and compare the findings with other chronic pain conditions such as neuropathic pain and headache; (ii) analyse the association between digital pain extent and psychological factors using longitudinal designs; (iii) test the role that different biopsychosocial factors may play as mediators/moderators of the association between digital pain extent and pain-related cognitions and emotions among individuals with chronic musculoskeletal pain; (iv) to explore differences and similarities between tools used to evaluate pain extent to determine the most appropriate tool for use in research settings.

### Limitations of the study

First, due to the cross-sectional nature of the current study, we are unable to establish any causal relationship between digital pain extent and the psychological factors assessed as well as pain intensity and pain-related disability. Second, most of the sample reported low back pain, and the results of the present study may underestimate the association between digital pain extent and psychological measures in other musculoskeletal pain locations. Third, other relevant protective psychological factors such as psychological flexibility, pain-related resilience, or sense coherence were not explored and could play a role in the association with digital pain extent. Fourth, persistent pain is often associated with comorbidities. We evaluated the presence or not of comorbidities in the present sample. However, the specific comorbidities that existed for each participant were not meticulously reported and therefore, they were not included in the data analyses. Fifth, the self-reported tool that was used to evaluate expectations of recovery was designed to assess expectations of treatment. This could explain the lack of association between expectations and digital pain extent. Sixth, a recent definition for chronic pain -chronic primary/secondary pain- has been published [48, 49]. This definition is later than our eligibility criteria process. We used the criteria proposed by the ACTTION-American Pain Society Pain Taxonomy [27]. However, some musculoskeletal clinical conditions that are considered in this novel definition may have been missed (i.e., persistent musculoskeletal pain related to disease of the nervous system -multiple sclerosis-) [49]. Finally, the evaluation method used in this study (digital pain extent), may limit replication of this study, as an electronic device (e.g., tablet) and a specific software (pain sketch in this case) are needed. Likewise, literacy with digital tools was not evaluated among patients, thus, this could introduce any bias in the study. This should inspire, as mentioned above, studies to determine the best tool to evaluate the presence of widespread pain and its location reported by patients with chronic pain.

## Conclusions

This cross-sectional study showed that a larger digital pain extent is associated with higher pain intensity in people with chronic musculoskeletal pain. However, the results did not support an association between digital pain extent and psychological measures as well as pain-related disability.

## Data Availability

All data relevant to the study are included in the article or are available are available from the corresponding author on reasonable request.

## Abbreviations

PROMs: Patient reported outcome measures
EXPECT: Expectations for Complementary and Alternative Medicine Treatments
GCPS: Chronic pain grade questionnaire
CPAQ: Chronic Pain Acceptance Questionnaire
PSEQ: Pain Self-Efficacy Questionnaire
LOT-R: Life Orientation Test-Revised
VIF: Variance inflation factor
